# Exosome–transmitted microRNA‐133b inhibited bladder cancer proliferation by upregulating dual‐specificity protein phosphatase 1

**DOI:** 10.1002/cam4.3263

**Published:** 2020-07-06

**Authors:** Xiaoxiao Cai, Lili Qu, Jian Yang, Junwen Xu, Li Sun, Xiaowei Wei, Xiaojun Qu, Tingting Bai, Zhirui Guo, Yefei Zhu

**Affiliations:** ^1^ Laboratory Medicine Center The Second Affiliated Hospital Nanjing Medical University Nanjing Jiangsu China; ^2^ Urinary Surgery The Second Affiliated Hospital Nanjing Medical University Nanjing Jiangsu China; ^3^ Gerontology Center The Second Affiliated Hospital Nanjing Medical University Nanjing Jiangsu China

**Keywords:** Bladder cancer, Dual‐specificity protein phosphatase 1, Exosome, microRNA‐133b, Proliferation

## Abstract

Bladder Cancer (BC) is the ninth most common tumor in the world and one of the most common malignant tumors of the urinary system. Some studies reported that miR‐133b expression is reduced in BC, but whether it plays a role in the development of BC and its mechanism is unclear. microRNAs can be packaged into exosomes to mediate communication between tumor cells, affecting their proliferation and apoptosis. The objective of this study was to investigate the effect of exosomal miR‐133b on BC proliferation and its molecular mechanism. Firstly, the expression of miR‐133b was evaluated in BC and adjacent normal tissues, as well as in serum exosomes of BC patients and healthy controls. Then the delivery and internalization of exosomes in cells was observed through fluorescence localization. Cell viability and apoptosis were assessed in BC cells transfected with mimics and incubated with exosomes. The role of exosomal miR‐133b was also analyzed in nude mice transplant tumors. Furthermore, the target gene of miR‐133b was predicted through bioinformatics. The level of miR‐133b was significantly decreased in BC tissues and in exosomes from serum of patients, which was correlated with poor overall survival in TCGA. Exosomal miR‐133b could be obtained using BC cells after transfection with miR‐133b mimics. The miR‐133b expression increased after incubation with exosomal miR‐133b, which lead to the inhibition of viability and increase of apoptosis in BC cells. Exosomal miR‐133b could suppress tumor growth in vivo. In addition, we found that exosomal miR‐133b may play a role in suppressing BC proliferation by upregulating dual‐specificity protein phosphatase 1 (DUSP1). These findings may offer promise for new therapeutic directions of BC.

## Graphical abstract

Upregulation of exosomal microRNA‐133b targeting by DUSP1 inhibited bladder cancer proliferation. It may be as a therapeutic opportunity for bladder cancer.

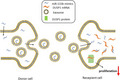

## 1 INTRODUCTION

Bladder cancer (BC) is the ninth most frequently diagnosed cancer worldwide,^1^ with about 549,393 new cases and 199,922 deaths in 2018.^2^ Muscle invasion occurs in about 20% of patients, and 7% of the non‐muscle invasive patients were at greater risk. However, the diagnosis and treatment methods of BC and the 5‐year survival remained unchanged.^3^ Thus, it is particularly important to investigate the mechanism of BC.

microRNAs (miRNAs) are tiny RNA molecules, 22‐25 nucleotides in length, that are involved in almost all known cancer regulatory processes. miR‐133b is a muscle‐specific miRNA that acts on cardiomyocytes and ion channel transmission by regulating downstream mediators.^4^ Additionally, low expression of miR‐133b can be used as a marker to evaluate the prognosis of BC patients and may be a target to suppress the proliferation of BC.^5^, ^6^, ^7^, ^8^ However, the transport mechanism of miR‐133b in BC cells needs further research.

Exosomes are 50‐150 nm extracellular vesicles that are present in nearly all body fluids. They stably carry various molecules (including miRNAs), which mediate signal transduction and play a major role in communication between cells.^9^, ^10^, ^11^ The exosomes carrying miRNAs secreted by tumor cells can be transferred into normal cells, making them prone to pathological proliferation.^12^ To date, accumulated evidences have shown that the effect of exogenous miRNAs in tumors through exosomal transport is similar to that of endogenous miRNAs.^13^ Additionally, exsomal miRNAs were found to suppress the levels of corresponding target genes in vivo.^14^ So, we speculate whether miR‐133b can act on BC proliferation through the exosome pathway.

DUSP1 is a mitogen and stress‐inducible dual‐specificity protein phosphatase, which is implicated in inactivating MAPK family isoforms,^15^ regulating cell proliferation and apoptosis, originally identified as a growth factor and stress‐inducible gene.^16^ Previous literature indicates that DUSP1 can inhibit cell proliferation by regulating the MAPK signaling pathway in tumors.^17^, ^18^ Upregulated DUSP1 levels result in the blockage of SAPK phosphorylation and the inactivation of the SAPK/JNK signaling pathway which inhibit cancer progression.^19^ Our study found that DUSP1 was a potential target gene of miR‐133b, whose promoter sequence is complementary to miR‐133b, increasing with the upregulation of miR‐133b.

In this study, we found that miR‐133b was significantly decreased both in BC tissues and exosomes from BC serum. Further studies showed that exosomal miR‐133b/DUSP1 pathway is a factor in BC proliferation, making it a potential therapeutic target for BC.

## 2 MATERIALS AND METHODS

### 2.1 Specimens and cell culture

Eleven paired human BC tumor and adjacent normal tissues were collected from the Second Affiliated Hospital of Nanjing Medical University. The specimens were collected during surgery and immediately frozen in liquid nitrogen. These patients include nine males and two females, whose average age was 66.7 ± 10.8. According to the invasive degree, all specimens are divided into pTa‐pT1 (eight) and pT2‐ pT4 (three). According to the clinical grade, they may be classified into I (eight), II (two) and III (one). Twenty serum specimens were obtained from patients diagnosed with bladder cancer, and 20 healthy serum specimens were obtained from those participating in the physical examination. The BC cell lines 5637 and T24 were purchased from the Chinese Academy of Sciences Cell Bank, Shanghai of China. 5637 and T24 cells were cultured in RPMI 1640 medium and Dulbecco's modified Eagle's medium (HyClone, Utah, USA) respectively, both supplemented with 10% fetal bovine serum (Gibco, NY, USA) and 1% penicillin/streptomycin (HyClone). Cultures were maintained in a humidified atmosphere of 5% CO_2_ at 37°C. Ethics approval was obtained from the Ethics Committee of the Second Affiliated Hospital of Nanjing Medical University and written informed consent was obtained from all patients and healthy controls.

### 2.2 TCGA database analysis of survival time

One hundred and thirty five cases of bladder epithelial cell carcinoma tissue samples miRNAs data and corresponding clinical information from the TCGA tumor database were confirmed and downloaded (as of 6 July 2018). Before analyzing survival time, we have standardized the miRNA data to exclude the interference of other factors. The median value of miR‐133b expression was used as the dividing line to divide patients into high and low expression groups for analysis. The “survival” package in R software was used to plot the Kaplan‐Meier curves for miR‐133b.

### 2.3 Exosome isolation and labeling

Exosomes were isolated from the cell culture media and serum with total exosome isolation reagent (Thermo Fisher Scientific, MA, USA). The concentration of exosomal proteins was quantified with a BCA Protein Assay Kit (Beyotime, Nanjing, China). The purified exosomes were incubated with 4 μM of PKH67 (MIDI67, Sigma ‐Aldrich, MO, USA) and excess dye was removed using a 100‐kDa filter (Amicon Ultra‐0.5 mL, Millipore, Darmstadt, Germany). These nuclei were then labeled with 4’,6‐diamidino‐2‐phenylindole (DAPI) according to the manufacturer's guidelines (Beyotime).

### 2.4 Transmission electron microscopy

Exosomes were loaded on a carbon‐coated electron microscopy grid, fixed with 2.5% glutaraldehyde solution for at least two hours, and then negatively stained with phosphotungstic acid for five minutes. Images were obtained by TEM (JEM‐1010, Jeol, Japan) after drying.

### 2.5 Western Blot

Protein was extracted from exosomes and cells using radioimmunoprecipitation assay (RIPA) buffer (Beyotime) containing Phenylemethanesulfonyl fluoride (Beyotime). A total of 20 μg of protein was separated by sodium dodecyl sulfate ‐ polyacrylamide gel electrophoresis (SDS‐PAGE) and transferred to polyvinylidene fluoride (PVDF) membranes. The membranes were incubated overnight with primary antibodies as follows: CD63 (Abcam, MA, USA), CD81 (Abcam), DUSP1 (Abcam), GAPDH (Abmart, Shanghai, China). The membranes were then incubated with secondary antibodies: anti‐mouse IgG (Abmart), anti‐goat IgG (proteintech Group, IL, USA).

### 2.6 RNA isolation and detection of miRNA and mRNA

Total RNA was isolated from tissues, cell lines and exosomes using Trizol® reagent (Invitrogen, CA, USA). Five picograms of cel‐miR‐39‐3p mimic (RiboBio, Guangzhou, China) was added as external control in serum. Total RNA was reverse transcribed into cDNA using the miRNA 1 st Strand cDNA Synthesis Kit (for miRNA) and HiScript^®^ II Q RT SuperMix for qPCR (for mRNA) (Vazyme Biotech Co. Ltd, Nanjing, China). The level of miRNAs and mRNAs was measured using the miRNA Universal SYBR^®^ qPCR Master Mix (Vazyme Biotech Co. Ltd) and the HiScript^®^ II Q RT SuperMix for qPCR (Vazyme Biotech Co. Ltd), respectively. U6 was used as an internal control in tissues and cell lines, and the cel‐miR‐39‐3p was used as the external control in serum for miRNAs. Bulge‐loop^TM^ miRNA qRT‐PCR Primer Sets (one RT primer and a pair of qPCR primers for each set) specific for miR‐133b, U6 and miR‐39‐3p were designed (RiboBio). Specific primer sets used for DUSP1 (forward: 5′‐GGGCAGAAGAGAAAGGACTCA‐3′, reverse: 5′‐CATTTG TGAA GGCA GAC AC CTA‐3′); and the relative gene expression was normalized to GAPDH (forward: 5′‐TGTGGGCATCAATGGATTTGG‐3′, reverse: 5′‐ACACCATGTATTCCGGGTCAAT‐3′). β‐actin was used as internal control. Relative gene expressions were analyzed by 2^△△ct^ method.

### 2.7 Cell transfection

RNA duplexes and luciferase reporter plasmid were chemically synthesized by GenePharma Company (Shanghai, China). The corresponding sequences are listed as follows: miR‐133b mimics: 5′‐UUUGGUCCCCUUCAACCAGCUA‐3′, NC mimics: 5′‐UUCUCCGAACGUGUCACGUTT‐3′. Lipofectamine 3000 reagent (Invitrogen) was used to transiently transfect the miRNAs into target cells according to the manufacturer's protocol.

### 2.8 Exosome treatment In vitro

BC cells were cultured with exosomes from relevant cells which were transfected with miR‐133b mimics or miR‐NC for 24 hours. These exosomes were named as miR‐133b‐EXO or NC‐EXO. Then 5637 and T24 cells were seeded into six‐well plates, and 80 μg/mL exosomes from different groups were added and incubated with the same type of cells.

### 2.9 Cell proliferation assay

The proliferative ability of BC cells was assessed using the cell counting kit‐8 assays (CCK8, Dojindo Laboratories, Kumamoto, Japan). 5637 and T24 cells (24 hours after transfection) were plated into the 96‐well plate at a density of 5000 cells/well and incubated for 0 hours, 24 hours, 48 hours and 72 hours, respectively. Subsequently, 10µL of CCK‑8 solution was added to each well for incubation at 37˚C for two hours. The optical density (OD) was measured at 450 nm by using a microplate reader at.

### 2.10 Colony formation assay

After transfection or incubation with exosomes for 24 hours, 5637 and T24 cells were seeded into 6‐well plates and cultured for two weeks until most of the colonies contained more than 50 cells. The colonies were fixed with methanol for 10 minutes and stained with 0.1% crystal violet for 15 minutes.

### 2.11 Cell apoptosis assay

BC cells (5 × 10^5^/well) were mixed with 500 µL binding buffer and stained using 5 µL Annexin V‐FITC and 5 µL Propidium Iodide (PI) (BD Biosciences, CA, USA) according to the manufacturer's instructions. Cells were incubated for 20 minutes after mixing, and flow cytometry analysis was carried out within one hour. These data were analyzed using FlowJo software (version 10, Ashland, OR, USA).

### 2.12 Xenograft model

Five‐week‐old male nude mice were randomly divided into four groups of five, and were subcutaneously injected with 1 × 10^7^ T24 cells. When tumors grew to 50 mm^3^, agomir‐NC and agomir‐miR‐133b were injected every three days, and different exosomes (10 μg) isolated from 5637 and T24 cells transfected with miR‐133b/ NC mimics were injected into the center of the tumor every two days. Tumor diameter was measured using digital calipers. The mice were sacrificed after three weeks, and tumor size and weight were measured.

### 2.13 Statistical analysis

All data were analyzed using GraphPad Prism 7 (GraphPad, CA, USA). Statistical analysis between two groups was performed using Student's t‐test, and analysis between multiple groups was conducted by one‐way analysis of variance (ANOVA) with the Bonferroni correction. Differences were considered statistically significant at *P* value < 0.05.

## 3 RESULTS

### 3.1 Expression of miR‐133b in BC tissues

**Figure 1:**
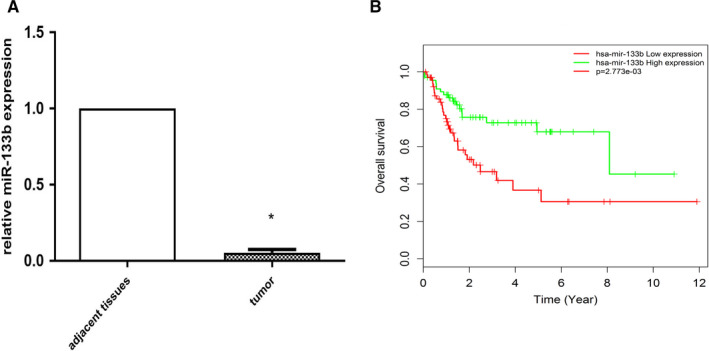
miR‐133b expression was significantly downregulated in BC tissues, and was correlated with poor overall survival in TCGA. Relative expressions of miR‐133b in BC tissues and adjacent normal tissues (A). BC patients with low miR‐133b expression had lower overall survival rates than patients with high miR‐133b expression in the TCGA cohort (B) (*P* < .001). *P < .05

The levels of miR‐133b in 11 BC specimens and their adjacent normal tissues were detected using qRT‐PCR. We observed significant downregulation of miR‐133b in BC specimens compared to normal tissues (Figure 1A). Moreover, the overall survival rate in the TCGA database decreased as the miR‐133b level was reduced. (Figure 1B).

### 3.2 Expression of exosomal miR‐133b in BC serum

**Figure 2:**
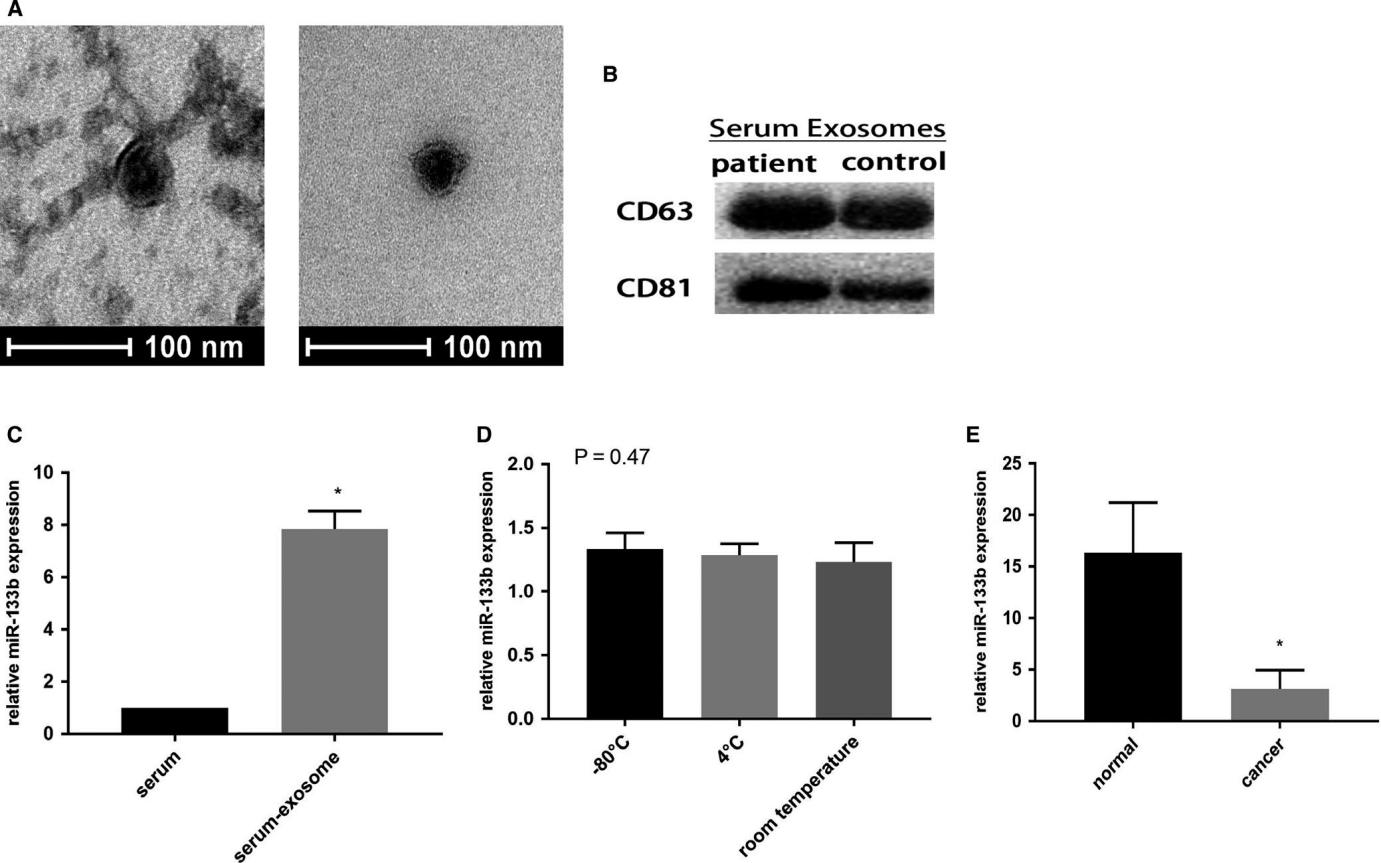
Expression of serum exosomal miR‐133b in patients with bladder cancer. Transmission electron microscopy image of exosomes derived from the serum of patients and controls. Scale bars represent 100 nm (A). Western blotting analysis showing the presence of CD63 and CD81 in exosomes (B). The expression of miR‐133b was detected in serum and serum exosomes (C). The relative expression levels of exosomal miR‐133b were stable after storing at −80°C, 4°C and room temperature for 12 hours respectively (D). qRT‐PCR detection of miR‐133b in exosomes from serum (E). *P < .05

Exosomes purified from the serums of patients with BC and healthy controls are similar to round particles (50‐150 nm, Figure 2A) according to our TEM analysis. Exosomes were further confirmed by two specific exosome markers CD63 and CD81 (Figure 2B). In view of the low level of miR‐133b obtained by direct extraction from serums of healthy controls, exosomal miR‐133b was easier to detect in BC serum (Figure 2C). Additionally, we found that the level of exosomal miR‐133b did not change clearly along with different temperature incubation conditions in serum samples of healthy controls (Figure 2D), indicating that miR‐133b was stable in exosomes from serum. Compared with the healthy control group, the expression of miR‐133b was significantly lower in exosomes from BC patients. (Figure 2E).

### 3.3 Effect of miR‐133b on BC cellular phenotype

**Figure 3:**
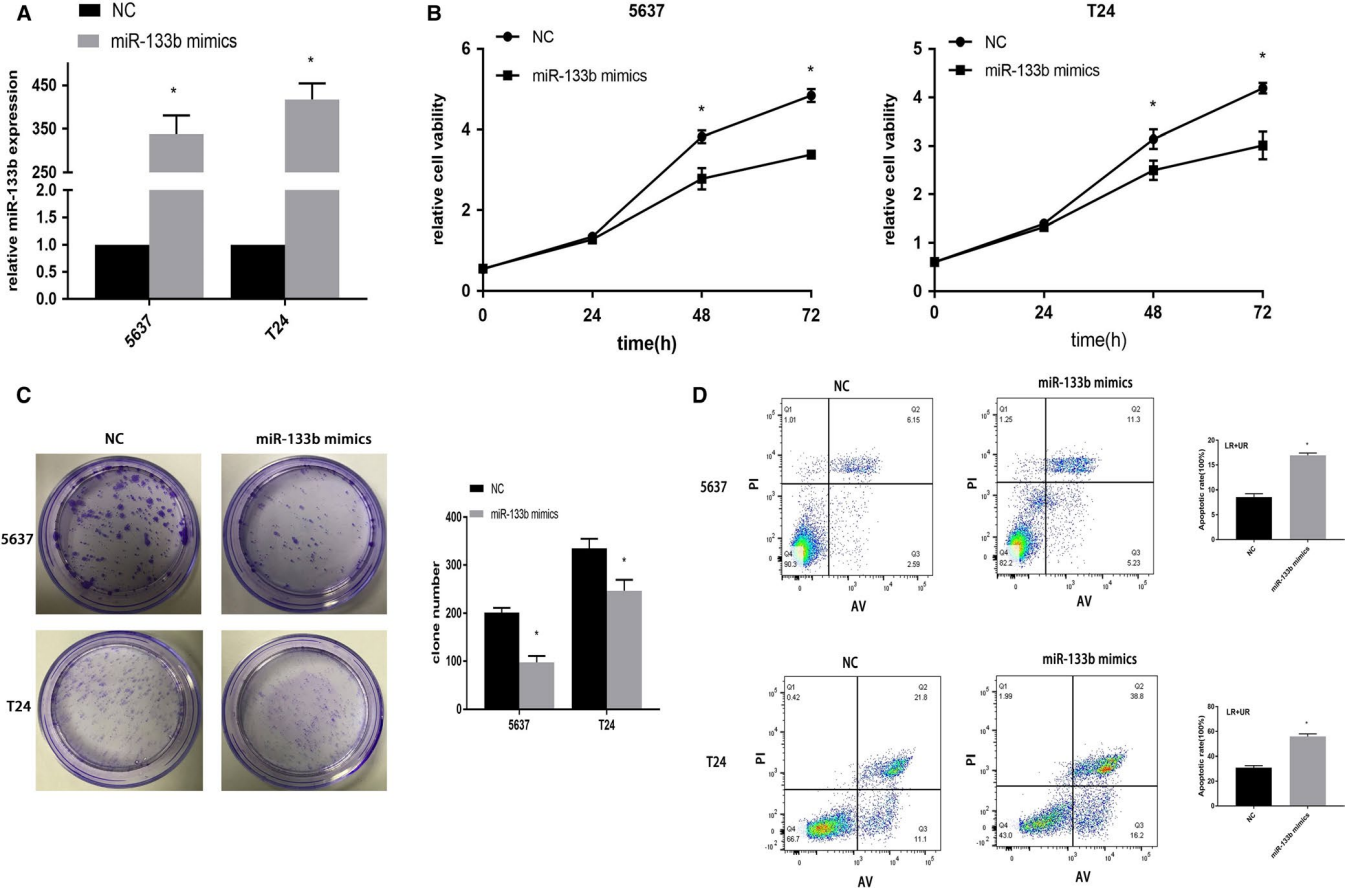
Effect of miR‐133b on bladder cancer cellular phenotype. 5637 and T24 cells were transfected with NC or miR‐133b mimics. The expression of miR‐133b in 5637 and T24 cells (A). A CCK8 assay detection of cell viability (B). Colony formation assays for evaluation of cell proliferation (C). Flow cytometry detection of the apoptosis of 5637 and T24 cells (D). *P < .05

To understand the biological role of miR‐133b in vitro, we transfected miR‐133b mimics and miR‐NC into BC cells, respectively. The expression of miR‐133b was remarkably upregulated in the miR‐133b mimics group (Figure 3A). Compared with the miR‐NC group, the proliferation of both BC cells was significantly suppressed after transfection with miR‐133b mimics after 48 hours (Figure 3B). Meanwhile, overexpression of miR‐133b strongly decreased the number of colonies in BC cells (Figure 3C). Additionally, flow cytometric analysis revealed that overexpressed miR‐133b induced apoptosis of BC cells (Figure 3D).

### 3.4 Exosomal miR‐133b mediates intercellular communication

**Figure 4:**
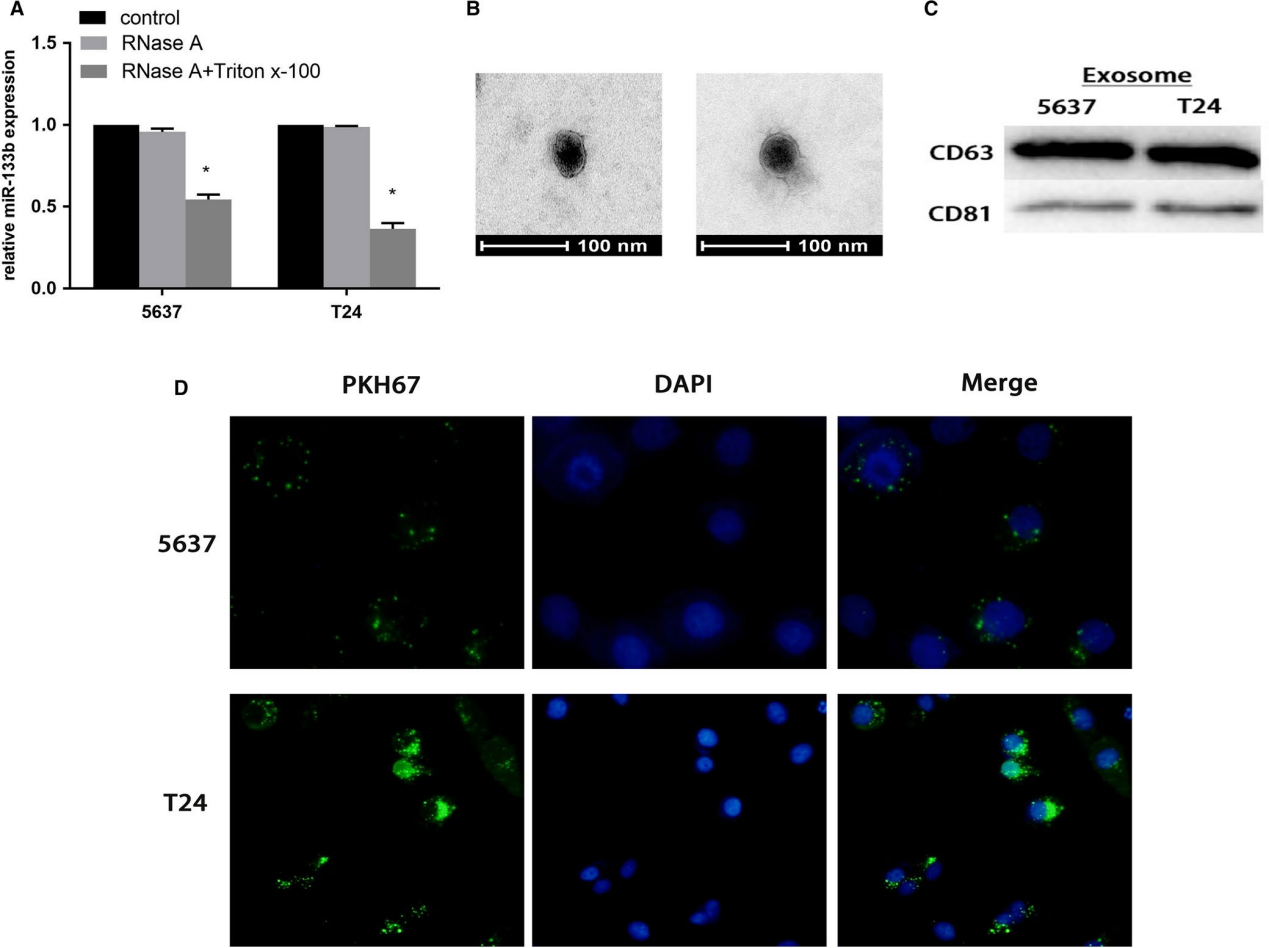
Exosomal miR‐133b act as a mediator for intercellular communication. The expression of miR‐133b in the medium of 5637 and T24 cells treated with RNase (3 μg/ml) alone or combined with Triton X‐100 (0.3%) for 20 minutes (A). Transmission electron microscopy image of exosomes derived from the serum of patients and controls. Scale bars represent 100 nm (B). Western blotting analysis showing the presence of CD63 and CD81 in exosomes (C). Confocal microscopy image showing the internalization of PKH67‐labeled exosomes derived from 5637 and T24 cells for three hours, green represents PKH67, and blue represents nuclear DNA staining by DAPI (D). *P < .05

In order to clarify the existence of extracellular miR‐133b, the medium of cells was treated with RNase and Triton X‐100. The level of miR‐133b did not change after treatment with RNase alone, but was significantly reduced after treatment with both RNase and Triton X‐100 (Figure 4A), indicating that extracellular miR‐133b was enveloped by the membrane rather than being released directly. The exosomes isolated from cells were identified by TEM and western blot (Figure 4B‐C). We then found that exosomes labeled with PKH67 were located in the cytoplasm of the recipient cells, indicating that exosomes were fused into recipient cells (Figure 4D).

### 3.5 Effect of exosomal miR‐133b on BC cellular phenotype

**Figure 5:**
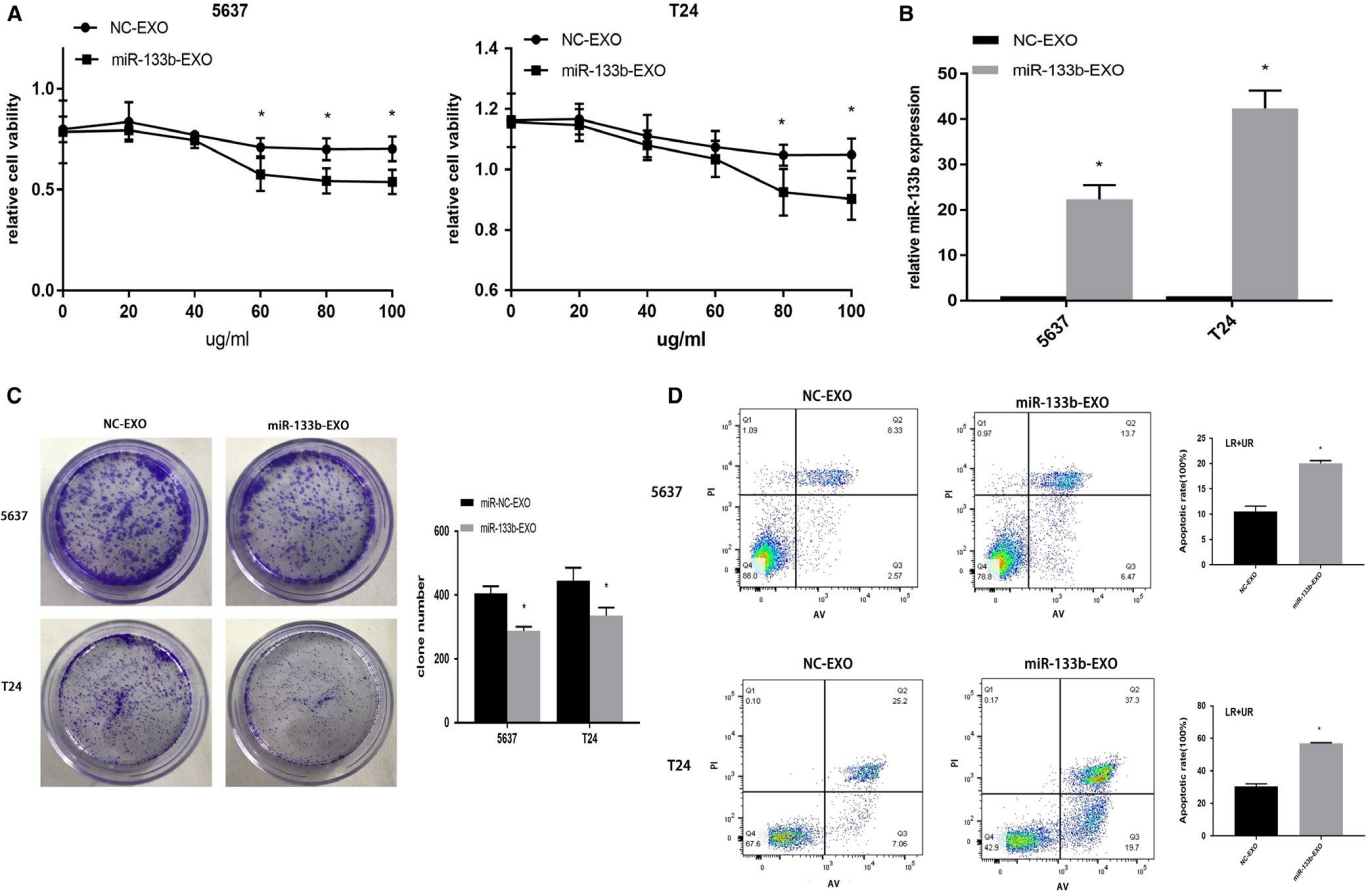
Effect of exosomal miR‐133b on bladder cancer cellular phenotype. Exosomes were isolated from 5637 and T24 cells transfected with NC or miR‐133b mimics, namely NC‐EXO and miR‐133b‐EXO, respectively. Their exosomes were extracted and cocultured with BC cells for 24 hours. A CCK8 assay detection of cell viability (A). The expression of miR‐133b in 5637 and T24 cells (B). Colony formation assays for evaluation of cell proliferation (C). Flow cytometry detection of the apoptosis of 5637 and T24 cells (D). *P < .05

We found that miR‐133b‐EXO could reduce the proliferation in a concentration‐dependent manner. When the treatment concentration of exosomes reached 80μg/mL, cell proliferation could be inhibited (Figure 5A). The results indicated that the expression of miR‐133b in BC cells incubated with miR‐133b‐EXO for 24 hours was significantly increased (Figure 5B). miR‐133b‐EXO reduced colonies (Figure 5C) and increased apoptosis of BC cells (Figure 5D).

### 3.6 miR‐133b regulates DUSP1 expression in BC

**Figure 6:**
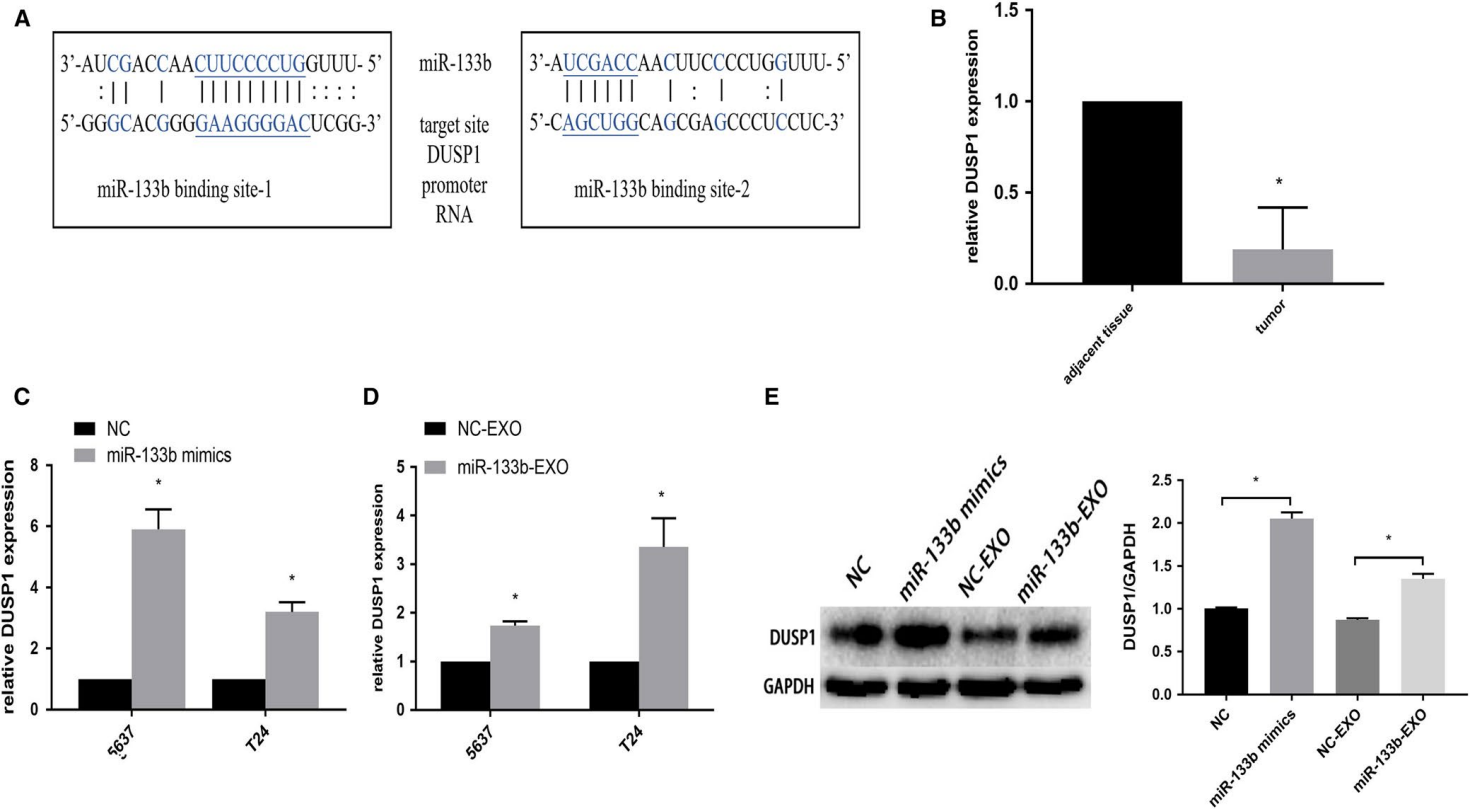
DUSP1 is a direct target of miR‐133b. The two target sites for miR‐133b within the DUSP1 promoter RNA (A). The expression of DUSP1 mRNA in BC tissues and adjacent normal tissues (B). miR‐133b upregulated the expression of DUSP1 in 5637 and T24 cells via transfection with miR‐133b mimics (C). DUSP1 mRNA was upregulated in 5637 and T24 cells after incubated with miR‐133b‐EXO (D). Western blots of DUSP1 in T24 cells were treated with NC or miR‐133b mimics and NC‐EXO or miR‐133b‐EXO (E). *P < .05

Dusp1 was predicted through bioinformatics. miR‐133b possessed sequence complementarity to two adjacent sequences within the promoter of DUSP1 (Figure 6A). Simultaneously, the expression of DUSP1 mRNA was higher in BC tissues than adjacent normal tissues (Figure 6B). DUSP1 mRNA was upregulated in BC cells after transfected with miR‐133b mimics or incubated with miR‐133b‐EXO (Figure 6C‐D). Furthermore, miR‑133b was positively correlated with DUSP1 protein expression in T24 cells (Figure 6E).

### 3.7 Effect of miR‐133b and exosomal miR‐133b on tumor in vivo

**Figure 7:**
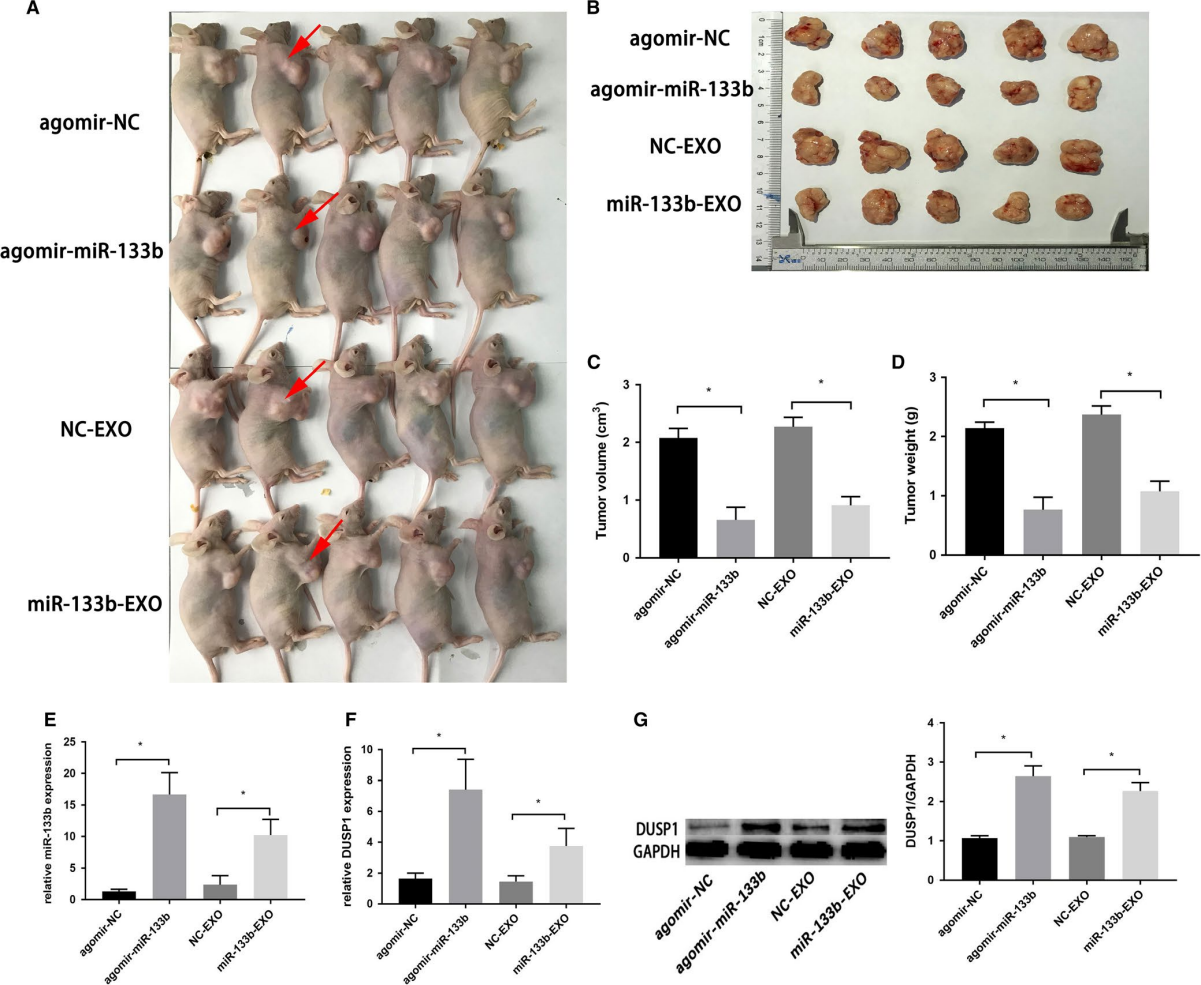
Effect of miR‐133b overexpression and exosomal miR‐133b on tumor in vivo. Xenograft model in nude mice. Red arrows show position of tumor (A). The excision tumors of T24 xenografts inoculated in agomir‐NC, agomir‐miR‐133b, NC‐EXO, miR‐133b‐EXO (B). The tumor volumes and weights of the agormir‐miR‐133b and miR‐133b‐EXO groups were significantly reduced compared to their counterparts in the control groups (C‐D). Detection of miR‐133b and DUSP1 mRNA expression in tumor tissues of nude mice treated with agomir‐NC, agomir‐miR‐133b, NC‐EXO, miR‐133b‐EXO by qRT‐PCR (E‐F). Western blots showed the expression of DUSP1 proteins in tumor tissues (G). *P < .05

We investigated the effects of miR‐133b and exosomal miR‐133b in BC growth in vivo (Figure 7A‐B). The average tumor weight and volume were significantly decreased in agomir‐miR‐133b group compared with agomir‐miR‐NC group. The same phenomenon was discovered in miR‐133b‐EXO group (Figure 7C‐D). As shown in Figure 7E, the expression of miR‐133b were increased in tumors after treatment with agomir‐miR‐133b and miR‐133b‐EXO. The expression of DUSP1 mRNA and protein was similarly upregulated in these two groups compared to their corresponding controls (Figure 7F‐G).

## 4 DISCUSSION

**Figure 8:**
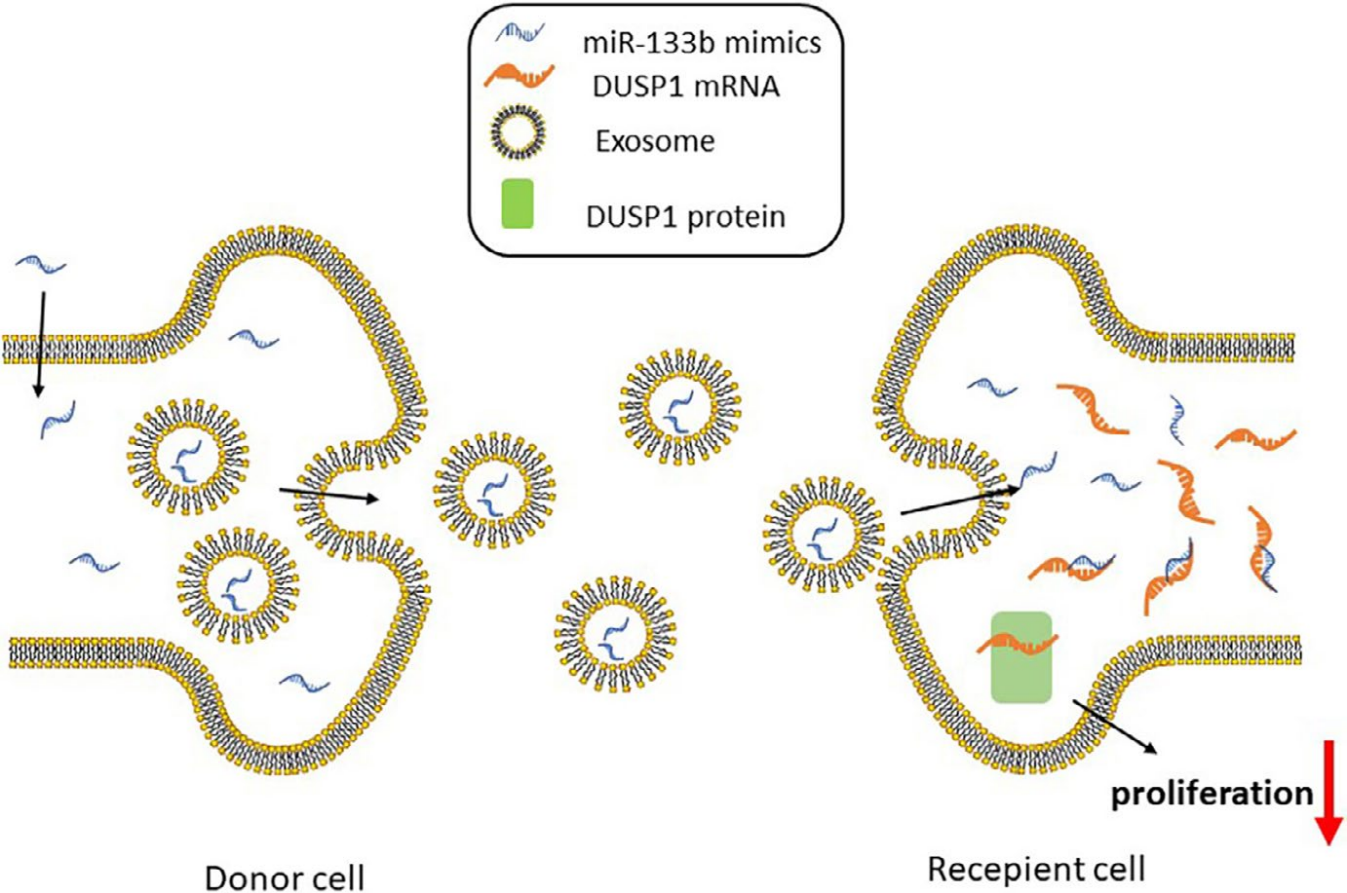
Schematic diagram of exosomal miR‐133b‐mediated BC proliferation. Exosomal miR‐133b derived from BC cells transfected with miR‐133b mimics, and then obtained from other BC cells again, to suppress the proliferation of BC by targeting DUSP1

BC is a complex urinary system malignancy with high morbidity and mortality rates.^20^ Although, molecular studies of BC have revealed new predictive biomarkers that may help guide treatment choices based on the underlying biology of the tumor over the years, the specific mechanism of BC occurrence is still unclear.^21^ In our study, miR‐133b was found significantly to be downregulated in BC tissues and exosomal miR‐133b was also lower in serums of BC patients. We found higher level of miR‐133b in serum exosomes than obtained by direct extraction from serum, which means that miR‐133b in serum mainly exists in exosomes, rather than in a free manner. Additionally, under different temperature incubation conditions, the expression of exosomal miR‐133b did not change significantly, indicating that miR‐133b was stable in serum exosomes. Exosomes packed with miR‐133b could be secreted from BC cells (donor cells) and taken up by BC cells (recipient cells) again. These exosomes significantly suppressed the proliferation of BC cells by targeting DUSP1 (Figure 8), inhibiting the growth of tumor in vivo.

Previous studies reported that miR‐133b is expressed in various cancers, such as renal, breast, prostate, colorectal cancers.^22^, ^23^, ^24^ We further found that overexpression of miR‐133b reduced cell proliferation and induced cell apoptosis in BC cells. Additionally, miR‐133b reduced the weight and volume of tumors in xenograft model. miR‐133b was reported to be downregulated in the tissues of BC patients and to inhibit tumor proliferation,^8^ which is consistent with our findings.

Posttranscriptional inhibition of miRNA binding to target mRNA 3'UTR is considered to be a typical pattern of miRNA‐mediated gene regulation. However, emerging evidence suggests that there are other regulatory models.^25^ The miRNAs directly bind to complementary DNA in the gene promoters to trigger gene expression, a phenomenon known as RNA activation (RNAa).^26^ The function of miRNAs in gene promoters is similar to a transcription factor for complementary motifs.^27^ For example, miR‐589 could induce the expression of cyclooxygenase‐2 (COX‐2) by recruiting a protein complex with transcriptional activators to the gene promoter.^28^ In our study, we found that the expression of the DUSP1 gene was promoted by the upregulation of miR‐133b both in vitro and in vivo. At the same time, preliminary analysis showed that there were complementary sequences between miR‐133b and DUSP1 promoter regions. We speculate that miR‐133b may induce DUSP1 expression by binding to the promoter region, and the specific mechanism remains to be further studied. DUSP1 can regulate cell cycle, apoptosis and autophagy through dephosphorylation of certain amino acids in MAPK subfamily members.^29^, ^30^, ^31^ Previous studies suggested that DUSP1 can participate in JNK pathway activation and affect apoptosis and play a tumor suppressor role through the MAPK pathway.^32^, ^33^

Uptake of exosomal miRNAs by cells further proves that extracellular miRNAs can be used as a signaling molecule to transfer from one cell to another, and mediate intercellular communication.^34^, ^35^ Using mesenchymal stem cells to support tumor‐targeted drug delivery, systemic administration of miRNAs‐enriched exosomes caused a significant reduction in cancer activity.^36^, ^37^ We found that the level of exosomal miR‐133b in serum was downregulated in patients with BC. Our results showed that exosomal miR‐133b repressed the proliferation of BC cells and induced apoptosis of BC cells by targeting DUSP1. Therefore, our results suggest that miR‐133b regulates the biological function of BC in vitro and in vivo through the exosomes targeting upregulation of DUSP1.

Compared with free miRNAs, the miRNAs in exosomes will hardly degrade in plasma, indicating that exosomal miRNAs can be used as a therapeutic tool.^38^ miR‐133b may be specifically encapsulated in large quantities with exosomes to provide certain basis for targeted therapy of BC. Utilizing the characteristics of miRNAs secretion, the exosomes containing specific tumor suppressor miRNAs for targeted tumor treatment could be designed.^19^ So, we can then take advantage of the miRNA secretion rules (such as the properties of miR‐133b) to target the patient and ultimately combination therapy, simultaneously targeting multiple miRNAs and/or proteins in further studies.

In conclusion, our findings provide robust evidence that exosomal miR‐133b functioned as a tumor inhibition factor targeting DUSP1 in BC proliferation. Furthermore, exogenous miR‐133b could be ingested by BC cells, and alleviate the malignant phenotype of BC cells in vitro and in vivo. Exosomal miR‐133b and/or other miRNAs will better characterize the disease and may enhance our ability at the individual level to bring about certain advances in the treatment of BC.

## CONFLICT OF INTEREST

The authors declare that they have no conflict of interest.

## AUTHOR CONTRIBUTION

Yefei Zhu conceived the idea and designed the experiment; Xiaoxiao Cai, Lili Qu and Jian Yang conducted experiments and data analysis; Junwen Xu, Li Sun, and Xiaowei Wei collected the specimens; Xiaoxiao Cai, Lili Qu wrote the manuscript; Xiaojun Qu, Tingting Bai, and Zhirui Guo revised the manuscript.

## Data Availability

The data used to support the findings of this study are available from the corresponding author upon request.
